# Assessment of Evaluation Tools for Integrated Surveillance of Antimicrobial Use and Resistance Based on Selected Case Studies

**DOI:** 10.3389/fvets.2021.620998

**Published:** 2021-07-08

**Authors:** Marianne Sandberg, Ayla Hesp, Cécile Aenishaenslin, Marion Bordier, Houda Bennani, Ursula Bergwerff, Ilias Chantziaras, Daniele De Meneghi, Johanne Ellis-Iversen, Maria-Eleni Filippizi, Koen Mintiens, Liza R. Nielsen, Madelaine Norström, Laura Tomassone, Gerdien van Schaik, Lis Alban

**Affiliations:** ^1^Department for Food Safety, Veterinary Issues and Risk Analysis, Danish Agriculture and Food Council, Copenhagen, Denmark; ^2^National Food Institute, Technical University of Denmark, Lyngby, Denmark; ^3^Department of Bacteriology and Epidemiology, Host Pathogen Interaction and Diagnostics Development, Wageningen Bioveterinary Research, Lelystad, Netherlands; ^4^Department of Infectious Diseases and Immunology, Utrecht University, Utrecht, Netherlands; ^5^Groupe de recherche en épidémiologie des zoonoses et santé publique, Université de Montréal, Saint-Hyacinthe, QC, Canada; ^6^UMR Astre, Cirad, INRAE, University of Montpellier, Montpellier, France; ^7^Veterinary Epidemiology, Economics and Public Health Group, Department of Pathobiology and Population Sciences, Royal Veterinary College, London, United Kingdom; ^8^Department of Farm Animal Health, Utrecht University, Utrecht, Netherlands; ^9^Unit of Animal Science and Unit of Social Science, Flanders Research Institute for Agriculture, Fisheries and Food (ILVO), Merelbeke, Belgium; ^10^Department of Reproduction, Obstetrics and Herd Health, University of Ghent, Ghent, Belgium; ^11^Department of Veterinary Sciences, University of Turin, Turin, Italy; ^12^Veterinary Epidemiology Unit, Department of Epidemiology and Public Health, Sciensano, Brussels, Belgium; ^13^Food and Agriculture Organization of the United Nations, Rome, Italy; ^14^Department of Veterinary and Animal Sciences, University of Copenhagen, Frederiksberg, Denmark; ^15^Department of Epidemiology, Norwegian Veterinary Institute, Oslo, Norway; ^16^Royal GD Animal Health, Deventer, Netherlands

**Keywords:** integrated surveillance, evaluation, tools, AMR, one health

## Abstract

Regular evaluation of integrated surveillance for antimicrobial use (AMU) and resistance (AMR) in animals, humans, and the environment is needed to ensure system effectiveness, but the question is how. In this study, six different evaluation tools were assessed after being applied to AMU and AMR surveillance in eight countries: (1) ATLASS: the Assessment Tool for Laboratories and AMR Surveillance Systems developed by the Food and Agriculture Organization (FAO) of the United Nations, (2) ECoSur: Evaluation of Collaboration for Surveillance tool, (3) ISSEP: Integrated Surveillance System Evaluation Project, (4) NEOH: developed by the EU COST Action “Network for Evaluation of One Health,” (5) PMP-AMR: The Progressive Management Pathway tool on AMR developed by the FAO, and (6) SURVTOOLS: developed in the FP7-EU project “RISKSUR.” Each tool was scored using (i) 11 pre-defined functional aspects (e.g., workability concerning the need for data, time, and people); (ii) a strengths, weaknesses, opportunities, and threats (SWOT)-like approach of user experiences (e.g., things that I liked or that the tool covered well); and (iii) eight predefined content themes related to scope (e.g., development purpose and collaboration). PMP-AMR, ATLASS, ECoSur, and NEOH are evaluation tools that provide a scoring system to obtain semi-quantitative results, whereas ISSEP and SURVTOOLS will result in a plan for how to conduct evaluation(s). ISSEP, ECoSur, NEOH, and SURVTOOLS allow for in-depth analyses and therefore require more complex data, information, and specific training of evaluator(s). PMP-AMR, ATLASS, and ISSEP were developed specifically for AMR-related activities—only ISSEP included production of a direct measure for “integration” and “impact on decision making.” NEOH and ISSEP were perceived as the best tools for evaluation of One Health (OH) aspects, and ECoSur as best for evaluation of the quality of collaboration. PMP-AMR and ATLASS seemed to be the most user-friendly tools, particularly designed for risk managers. ATLASS was the only tool focusing specifically on laboratory activities. Our experience is that adequate resources are needed to perform evaluation(s). In most cases, evaluation would require involvement of several assessors and/or stakeholders, taking from weeks to months to complete. This study can help direct future evaluators of integrated AMU and AMR surveillance toward the most adequate tool for their specific evaluation purpose.

## Introduction

The importance of combatting antimicrobial resistance (AMR) was highlighted in the Global Action Plan (GAP) released by the World Health Organization (WHO) in 2015 (1). It was further adopted by the Tripartite Collaboration consisting of the members of the WHO, Food and Agriculture Organization of the United Nations (FAO), and the World Organization for Animal Health (OIE) and endorsed by political leaders and the United Nations (UN) General Assembly (2). The Tripartite Collaboration acknowledges that the AMR challenge needs to be addressed using a One Health (OH) approach to reflect that the development and spread of AMR do not respect boundaries between sectors and, therefore, require cross-sectoral collaboration and prevention activities. One of the main objectives of the GAP is to initiate and maintain cost-effective integrated surveillance of antimicrobial use (AMU) and AMR at the global and national levels (1).

Ideally, combatting AMR requires engagement from actors within all sectors of animal health, food safety, environmental protection, plant health, and human health (3). All sectors need to be involved in surveillance to identify emerging resistance, understand the AMR epidemiology, and develop effective policies for AMU and AMR reduction. In short, the integration of sector activities and robust collaboration are essential for successful surveillance and control of AMU and AMR. According to Stärk et al. (4), OH surveillance describes the systematic collection, validation, analysis, interpretation of data, and dissemination of information collected in humans, animals, and the environment to inform decisions for more effective, evidence-based interventions. AMR genes are present in bacteria and spread among humans, animals, and the environment. A program of integrated surveillance of AMR in foodborne bacteria includes coordinated sampling and testing of antimicrobial susceptibility of bacteria from food-producing animals, food, and humans using epidemiological (including sampling) and microbiological methods that enable comparisons of results. The use of comparable methods is necessary to allow comparison of antimicrobial susceptibility results between different areas, countries, and regions (5, 6). Currently integrated OH AMU and AMR surveillance and monitoring systems exist or are under development in many countries (4). However, the surveillance programs do not always address all necessary sectors and they are rarely fully integrated (7). An integrated approach provides a better understanding of the epidemiology of AMR and an easier identification of the best intervention points and enhances the timeliness of surveillance by providing early warning of emergence of new resistant strains from one sector to another. Furthermore, a cross-sectoral collaboration may lead to knowledge/resource sharing, expertise exchange, and capacity building (8), which may result in cost savings and create more efficient and effective systems (9). Full integration might not be necessary to achieve the wanted outputs, and integration and collaboration in itself can be costly without always improving outputs (7, 10). A surveillance approach implies planning, data collection, analysis, interpretation, and dissemination of a given activity. It is useful to apply collaboration across different surveillance activities and integration in all or some of the activities. Identification of the optimal levels of integration to obtain the information needed for decision making is an important task in OH surveillance systems (7, 10).

Aenishaenslin et al. (7) suggested that the value of OH surveillance for AMR can be conceptualized and measured across a selection of different outcomes that can be classified in three dimensions, namely, (i) immediate, (ii) intermediate, or (iii) ultimate. Immediate outcomes include increased understanding of the AMR epidemiology at the human, animal, and environment health interface, and the value would lie in the intellectual or social capital generated. Intermediate outcomes include changes in policy or behaviors, and the expected value is the reduction in AMU and AMR that results from these changes. Ultimate outcomes include tangible benefits such as improved animal, human, and environmental health and associated socioeconomic benefits.

Apart from appropriate planning and designing, surveillance programs also need regular evaluation to remain operational, efficient, and cost-effective. Moreover, evaluation is needed to ensure that the goal is underpinned by the ongoing activities and shared with the essential stakeholders (11). Evaluation is complex and requires agreement on an evaluation objective, a process usually led by food safety/health authorities in consultation with other stakeholders. Secondly, an appropriate evaluation tool should be selected, which requires expertise and knowledge of surveillance evaluation.

Existing tools for evaluation of surveillance [e.g., (12, 13)] are not necessarily appropriate for integrated surveillance as they might not address aspects such as collaboration across sectors (12, 14). Characteristics of OH surveillance programs have been described, and recently, tools to evaluate integrated surveillance systems have emerged, targeting different aspects of the OH or other integrated surveillance activities (7, 11, 15–19). A tool may have been made for evaluation of a particular type of surveillance system, such as animal health surveillance. Still, it might also be used to assess other types of surveillance systems such as AMR surveillance, covering aspects such as sampling strategies and sample sizes of surveillance protocols. The latter may not be covered in details by the tools developed specifically for AMR surveillance evaluation. The different tools vary in their approaches, layouts and user-friendliness, comprehensiveness, terminology, aspects covered, capacity, training, and resources required to use them, as well as their specific usefulness for the evaluation of AMU and AMR surveillance. Hence, a characterization and meta-evaluation of the existing evaluation tools are called for to provide guidance on how to identify the best match between the evaluation objective, the resources available, and the selected evaluation tool.

During 2019–2020, an international network of scientists in the project “Co-Eval-AMR—Convergence in evaluation frameworks for integrated surveillance of AMR” (20) developed guidance for choosing an assessment approach from an inventory of tools suitable for evaluating integrated AMU and AMR surveillance systems, according to the needs of the users. The results presented here originate from the Co-Eval-AMR network aiming to guide assessors in their future selection of evaluation tools. A pilot version of the present study, using one surveillance system case and the first version of the assessment criteria, was published by Nielsen et al. in 2019 (21). The objective of the present study was to describe and assess the characteristics, functionalities, and suitability of tools that might be used for evaluation of integrated AMU and AMR surveillance.

## Materials and Methods

### Overview of the Evaluation Tools

In the following section, the six tools used are presented in brief.

#### Assessment Tool for Laboratories and AMR Surveillance Systems

The Assessment Tool for Laboratories and AMR Surveillance Systems (ATLASS) is a tool designed by the FAO for assessing and defining targets to improve national AMR surveillance systems in the food and agriculture sectors (18). It is composed of two modules: a surveillance module and a laboratory module. Each module includes two standardized questionnaires, which are to be completed by the assessors. The assessments generate a baseline and classify a “stage” for AMR laboratory capacity detection, AMR surveillance, and dissemination of information.

#### Evaluation of Collaboration for Surveillance

The Evaluation of Collaboration for Surveillance (ECoSur) tool aims at evaluating the organization, functioning, and functionalities of collaboration taking place in a multi-sectoral surveillance system (11). The final purpose is to assess whether collaboration as planned and implemented is relevant and functional to produce the expected collaborative outputs. The tool relies on the scoring of 22 attributes and three indexes characterizing the organization of collaboration at the governance and operation level and nine attributes referring to core functions of collaboration to ensure the sustainable operation of an effective multi-sectoral surveillance system. Three automatically generated outputs display the evaluation results for attributes and indexes and support the identification of strengths and weaknesses of collaboration and the formulation of recommendations for its amelioration.

#### Integrated Surveillance System Evaluation Project

The AMR integrated surveillance system evaluation project (ISSEP) tool is a conceptual tool developed in Canada with the aim to structure an evaluation of the added value of integrated surveillance systems for AMR (7). It comprises five evaluation levels that target the evaluation of OH integration in the surveillance system; its capacity to produce integrated information and expertise, to generate actionable knowledge, and to influence decision making; and health and economic impacts. For each level, a set of evaluation questions are defined, and links are made with existing evaluation tools. A semi-quantitative scale is applied to show the level of integration of the surveillance system (19).

#### Network for Evaluation of One Health

The Network for Evaluation of One Health (NEOH) tool is part of a framework resulting from the EU COST Action “Network for Evaluation of One Health” to provide science-based guidance for the evaluation of One Health and other integrated approaches to health (16, 20, 21). There are four elements, namely, “system definition and description of OH initiative within the system,” “theory of change” (ToC), “assessment of OH-ness,” and “outcome evaluation.” Qualitative assessment as well as semi-quantitative scorings are used for the evaluation of the degree and of the “OH-ness” (OH index and OH ratio) and metrics for different outcomes. Illustrative web diagrams of the distribution of scores for gap identification are presented in the Excel tool for assessment of OH-ness (20).

#### Progressive Management Pathway Tool for AMR

The Progressive Management Pathway tool for AMR (PMP-AMR) tool is a self-assessment tool designed by the FAO to provide guidance to countries for implementation of their National Action Plans (NAP) for AMU and AMR (17, 21). It includes four focus areas for evaluation: awareness, evidence, governance, and practices. For each focus area, specific activities, achievements, and key performance indicators (KPI) are listed. The tool provides a dashboard, showing the progress made for each focus area toward an optimal and sustainable use of antimicrobials.

#### Survtools

SURVTOOLS was developed as a part of the EU FP7-funded project RISKSUR: risk-based animal health surveillance systems. The evaluation tool (EVA tool) is a support tool for the evaluation of animal health surveillance systems, developed to provide guidance for evaluation of animal health surveillance including economic evaluation (12, 21). When planning an evaluation, the user is guided through three main steps: defining the evaluation context; defining the evaluation question; and selecting the evaluation attributes and the economic criteria. Furthermore, the tool provides additional information and guidance on how to use the evaluation plan to perform the evaluation and how to report on the evaluation outputs. An online web version of the EVA tool is available (12).

### Methodology Used to Assess the Tools

The details of the scoring scheme for functional aspects, the SWOT-like approach, and the scoring scheme for the themes describing the scope of the tools are presented below.

#### The Case Study Approach

A total of eight country-based case studies of AMU and AMR surveillance systems were included in the study (Table 1). Each country-based case study was undertaken by individuals or a group of individuals with expertise on the respective national cases (hereafter called the assessors), making a total of 20 assessors. The choice of case was the NAP on AMR or parts of it in the respective assessor's country. To collect the information needed to carry out the assessment, the assessors reached out to additional experts and other sources.

**Table 1:** Overview of eight country-based case studies involving six different tools for evaluation of surveillance of antimicrobial use and resistance, 2019.

**Country**	**Tools**	**Name of surveillance program**	**Component(s) covered**
Belgium	PMP-AMR and NEOH	Belgian AMR Surveillance Programme (as suggested in the Belgian National Action Plan)	Swine, veal calves, poultry (broilers/laying hens), and humans
Denmark	PMP-AMR, ATLASS, ECoSur, NEOH, and SURVTOOLS	Danish Integrated AMR Surveillance Programme (DANMAP)—selected parts	Pigs
Canada	ISSEP	Canadian Integrated Program for Antimicrobial Resistance Surveillance (CIPARS)	Humans, livestock, and food chain
Italy	NEOH, PMP-AMR, and SURVTOOLS	Italian ClassyFarm Surveillance Programme (data from the Piedmont region)	Pigs
Norway	PMP-AMR and NEOH	NORM-VET monitoring program for antimicrobial resistance in the veterinary and food production sectors (NORM-vet)	Broilers
The Netherlands	SURVTOOLS and NEOH	Monitoring of Antimicrobial Resistance and Antibiotic Usage in Animals in the Netherlands (MARAN)	Broilers, slaughter pigs, veal calves, and dairy cows
United Kingdom	ISSEP	Surveillance of AMU and AMR in the United Kingdom	Humans, livestock, and food chain
Vietnam	ECoSur	Surveillance of AMR in Vietnam	Humans, food products, and animals

*ATLASS, the Assessment Tool for Laboratories and AMR Surveillance Systems; ECoSur, Evaluation of Collaboration for Surveillance tool; ISSEP, integrated surveillance system evaluation project; NEOH, Network for Evaluation of One Health; PMP-AMR, The Progressive Management Pathway tool on AMR; AMU, antimicrobial use; AMR, antimicrobial resistance*.

The assessors met regularly, and initially, there was an assessment methodology developed in collaboration with selected members of the Co-Eval-AMR network group. The methodology included two standardized scoring schemes; a strengths, weaknesses, opportunities, and threats (SWOT)-like analysis scheme; and templates for reporting and instructions. The evaluation tools were applied on the country-based case studies using one or more tools on each case. Overall, the outcome was the users' experience regarding applicability of the tool. Each tool was assessed between one and four times.

#### Scoring Functional Aspects

A scoring scheme aiming at assessing 11 functional aspects was developed, and answers were scored numerically, where 1 = not covered, 2 = not well-covered, 3 = more or less covered, and 4 = well-covered. With each score, a comment was requested explaining the score. The 11 aspects were as follows: (1) user friendliness, (2) compliance with evaluation objectives, (3) efficiency (number of people and time taken vs. what the evaluation should be used for), (4) use of a step-wise approach to the evaluation, (5) overall appearance, (6) generation of actionable evaluation outputs, (7) evaluation of OH aspects, (8) workability in terms of required data, (9) workability in terms of required people to include, (10) workability in terms of analysis to be done, and (11) time taken for application of the tool.

The combined scores for each tool were presented in a heat map. In the case one assessor/assessor group scored over a range of numbers, averaging was used followed by rounding up if necessary to obtain a whole number for the total score. A crude summary score for each tool was calculated and presented in heat maps. The scores should only be interpreted relatively within this study material. The justification for each score, provided by the individual assessors, was condensed by the first author and checked for correctness by the other authors, and the “condensed results” were then presented.

#### A SWOT-Like Approach

A SWOT-like scheme was developed asking the assessors to answer four questions: (1) things that I liked or that the tool covered well; (2) things that I struggled with when using this tool; (3) things people should be aware of when using this tool; and (4) things that this tool covers insufficiently. A qualitative synthesis of the result was done in two steps. First, all individual phrases were captured. In a second step, phrases with the similar meaning were reduced into one, implying that a phrase was simplified or made into one word, if possible. It also implied that no phrase or word was repeated for each of the SWOT analyses and tools. The first synthesis was carried out by the assessors for the tools they had applied. The second synthesis was condensed by two of the authors, and the condensed results were checked for correctness by the other authors and subsequently presented.

#### Scoring Themes for the Scopes

A second scoring scheme consisted of eight themes to describe the scope of the tool: developed specifically for AMU and AMR, collaboration, resources, output and use of information, integration, governance, adaptivity, and technical operations. Seven of the themes included in the scheme were developed in the Co-Eval-AMR project (22). Additionally in this study, the theme governance was added. The objective was to score how well each theme was covered by the specific evaluation tool. A more detailed description of the individual theme scope is given in Table 2. The same scoring scale as in the Scoring Functional Aspects section was used. The combined scores for each tool were presented in a heat map, based on a similar way of estimation as described in the Scoring Functional Aspects section. A crude summary score for each tool was calculated, but this should only be interpreted relatively within this study material. Again, the free text justifications behind the scores provided by the assessors were synthesized by the first author, checked for correctness by the other authors, and subsequently presented.

**Table 2:** Description of the themes describing the scope of the tool in relation to surveillance identified in the Co-Eval-AMR project and used for the additional assessment of the evaluation tools for surveillance programs/activities.

**Theme**	**Description of the themes**
AMU and AMR	Questions that are specifically addressing the case of AMR (occurrence, prevention, or response) or AMU (recording and management)
Collaboration	Questions on the framework of collaboration (organization of roles and responsibilities) and the object of collaboration (exchange of data, information, and knowledge and sharing of capacities). This category also covers questions about the inclusive participation of stakeholders (e.g., considering gender)
Resources	Questions quantitatively addressing human, physical, and financial resources. Questions on the training level of human resources are also considered in this category
Output and use of information	Questions on surveillance outputs that are provided to inform public and private stakeholders, their use to inform decision making, and the benefits from this use (expected, perceived, or measured)
Integration	Questions considering three levels of integration: • integration of data systems (within organizations and at national, regional, or international level; data systems interoperation; and adherence to international testing and data standards) • integration between sectors and disciplines (knowledge integration, shared decision making and planning, and formulation of common goals) • integration in the national and international context motivating the need for surveillance (link to decision making, shared decision making, and planning between countries)
Governance^*^	Questions related to the legislative framework as well as the steering and coordinating mechanisms for the surveillance system: legislation, steering, and criteria (limits and goals for reduction)
Adaptivity	Questions on any structural elements allowing for the surveillance system to adapt and evolve. This may include not only tools, plans, and agreements to evolve (e.g., continuous learning programs and external evaluation) but also the features of management and governance allowing for regular evaluation and adaptation of operations (e.g., frequency of meeting and regularity of progress reports)
Technical operations	Questions on technical features of surveillance operations (surveillance design, laboratory capacities, management of specimens, tests applied, data management, and analysis), their quality management (SOP, traceability), and the assessment of their performance (sensitivity and specificity)

* *Governance was included as a separate theme in this study but is not a separate theme on the https://guidance.fp7-risksur.eu/welcome/decision-support/*.

## Results

All detailed answers and justifications from the scoring of the functional aspects and the themes and from using the SWOT-like approach are published on the Co-Eval-AMR project webpage (https://coevalamr.fp7-risksur.eu/) and in Nielsen et al. (21).

### Scoring of the Functional Aspects of the Tool

The results from the scoring of the case studies according to the 11 functional aspects of AMU and AMR surveillance systems are shown in Table 3. A summary of the justifications behind the scores is shown in Table 4. A crude summary of the scores showed that ISSEP and NEOH had the lowest scores, 25 and 30 respectively of the total 44 that could have been achieved. ATLASS and PMP-AMR had the highest, 39 of the 44 possible.

**Table 3:** Result of the scoring of all six tools with respect to the 11 functional aspects, shown as a heat map (the number of times the tool was assessed is given in the bracket). The scoring scale used was where 1 (red) = not covered, 2 = not well covered (orange), 3 = more or less covered (yellow), 4 = well covered (green).

	**ISSEP (2)**	**ECoSur (2)**	**ATLASS (1)**	**PMP–AMR (4)**	**NEOH (5)**	**SURVTOOLS (2)**
User friendliness	2	3	4	4	2	4
Meets evaluation needs/requirements	3	4	2	3	4	3
Efficiency	2	4	4	4	3	3
Use of a step-wise approach to the evaluation^*^	3	2	4	4	3	2
Overall appearance^**^	2	3	4	4	2	4
Generation of actionable evaluation outputs	2	4	4	4	3	2
Allows evaluation of one health aspects	3	3	4	2	4	2
Workability in terms of required data (1: very complex and 4: simple)	2	3	1	4	2	3
Workability in terms of people to include (1: many and 4: few)	2	3	4	3	2	4
Workability in terms of analysis to be done (1: difficult and 4: simple)	2	4	4	4	3	3
Time taken for application of tool: time (1: >2 months, 2: 1–2 months, 3: 1 week−1 month, and 4: <1 week)	2	3	4	3	2	3
Crude summary score	25	36	39	39	30	33

* *Only scored by 11 of the 20 of the assessors*.

** *Only scored by one of the two assessors of ISSEP. The scoring scale used was as follows: 1 = not covered (red), 2 = not well-covered (orange), 3 = more or less covered (yellow), and 4 = well-covered (green)*.

**Table 4:** Results of synthesis of the underlying reasoning for the scoring according to the 11 functional aspects.

	**ISSEP (CA and UK)**	**ECoSur (VN and DK)**	**ATLASS (DK)**	**PMP-AMR (BE, DK, IT, and NO)**	**NEOH (DK, BE, IT, NO, and NL)**	**SURVTOOLS (DK and NL)**
User friendliness	Conceptual framework easy to follow. Evaluation(s) more complicated	Relatively easy to understand and could be improved with a web interface	Can be used without much preparation	Easy to understand and fill in without training	Complex without training, long/exhausting. Scoring OH attributes is relatively simple	Tool itself is easy to fill in, but more complex to conduct evaluations
Meets evaluation needs/requirement	Relationships of integrated surveillance activities/outputs described. No guidance on evaluation	Measurement of the level of collaboration, but not the overall added value of collaborating for surveillance activities	Predefined network is comprehensive, but measurement of smaller progressions not possible	Qualitative scoring system could be improved. Partially meeting needs for AMU and AMR evaluation(s)	Comprehensive, less intuitive to use for specific technical details/laboratory part	Epidemiological performance easiest to perform, other parts more difficult
Efficiency	Requires a lot of time to conduct evaluation(s)	Evaluation matrix easy to understand/apply. Validation meeting with stakeholder required	Questionable whether all data are really needed	Easy to fill in. Immediate generation of results. Suitable for administrators	Takes a long time to fill in tool. “Theory of change” (ToC) could be better integrated. Not a management tool	Takes some time to fill in the tool and longer time for evaluations
Use of a step-wise approach to the evaluation	The tool has five evaluation levels	Only possible to follow progress of collaboration if evaluation repeatedly done	Follows a step-wise approach with areas containing sub-categories reflecting the level of implementation and geography	Follows (inherent) a step-wise approach with four levels with logic progression. Level 1: planning of activity/locally and levels 2, 3, and 4: undertaking activities /regionally/nationally	Stepwise approach to evaluation with the following steps: context description, initiative within context description, OH-ness, and ToC (outcome and impact). If evaluation of progress, repeated evaluations over time needed	Does not follow a step-wise approach. Order would be given by choice of evaluation question(s) and not by the toll itself
Overall appearance	The conceptual framework is well-presented	Well-structured, web platform needed	Useful for evaluation of AMU and AMR and residue surveillance at laboratory level	The general assessment part excellent, the sector specific less so. Nice layout, some parts could be improved	Extensive handbook. Excel tool is mostly understandable but too compressed in layout	Generates evaluation plan. Takes time to evaluate integrated surveillance. Objective results
Actionable evaluation outputs	No clearly defined actionable outputs	Generation of three graphical outputs of results: one for organizational attributes, one for organizational indexes, and one for functional attributes	Monitors progress and suggests next level	Actions can be agreed upon during assessment. Graphics could be improved. Gaps in sector evaluation	A web diagram makes it easy to identify gaps. Scoring is subjective: may lead to biased results	Not generated by tool. Evaluation could generate first-level actionable outputs (e.g., effect of designs). Other outputs on, e.g., awareness more difficult to obtain
Evaluation of OH aspects	Comprehensive	Existence of specific attributes measuring OH aspects, e.g., shared leadership	All sectors covered and measures integration	Not addressed in particular	Major strength of the system's approach and the tool	Can be used for all aspects. Layout does not support all components
Workability regarding required data (1: very complex and 4: simple)	Large amounts of data required	Dependent on the complexity of the surveillance system evaluated	Large amounts of data required	Apparently simple. Data are easily accessible	Requires effort/time to gather data. Some data complex to get (e.g., learning/system organization)	Relatively simple to get the data for filling in tool, but for some evaluation questions/objectives, it is complex to acquire the data
Workability regarding required people (1: many and 4: few)	Stakeholders from all sectors required	Meant to be applied by an evaluation team	Needs expertise from several areas	All stakeholders invited to evaluation meetings (2 days). One person can do evaluation, but then data capture needed (e.g., through interviews)	Interview of essential actors and stakeholders, but only one evaluator needed	Few people needed
Workability regarding analysis to be done (1: difficult and 4: simple)	No guidance on analysis provided	Easy identification of the criteria influencing the evaluation results to support formulation of recommendations	Automated analysis	Generated by the tool. Mostly yes/no answers to questions	Once tool is filled in, it provides support for analyses. Comparing ToC and scoring difficult	Dependent on the number and complexity of evaluation question(s)
Time (1: >2 months, 2: 1–2 months, 3: 1 week−1 month, and 4: <1 week)	Long time required for evaluation(s)	Dependent on the complexity of the surveillance system evaluated	If assessor experienced in surveillance or detailed NAP report available, takes relatively short time	Take relatively short time	Filling in the Excel tool is relatively fast once you have the information ready. Defining the ToC and gathering data is time-consuming	Short time to fill in tool. Long time for some of the evaluation objectives/questions

For OH aspects, ATLASS and NEOH scored the highest. PMP-AMR, ATLASS, ECoSur, and NEOH provide semi-quantitative scores for the aspects evaluated, whereas ISSEP and SURVTOOLS will result in a plan for how to conduct evaluation(s). ISSEP, ECoSur, NEOH, and SURVTOOLS allow for in-depth analyses and, therefore, require more complex data, information, and specific training of the evaluator(s). PMP-AMR and ATLASS seemed to be the most user-friendly tools, particularly designed for food safety authorities managing the surveillance system.

### The SWOT-Like Approach

The results of the SWOT-like approach applied to assess the tools are shown in Table 5. The variation in answers to the four SWOT-like questions was low among the assessors of each tool, indicating consistency regarding the general impression of the tools. The PMP-AMR and ATLASS were liked for the semi-quantitative scorings that could be made directly and that the tools were particularly made for evaluation of AMR surveillance systems. What is not covered in these two tools is the environmental, plant, and human parts of surveillance.

**Table 5:** Synthesis of phrases provided in the SWOT analysis of six different evaluation tools used in eight country-based case studies.

	**ISSEP (CA and UK)**	**ECoSur (VN and DK)**	**ATLASS (DK)**	**PMP-AMR (BE, DK, IT, and NO)**	**NEOH (DK, BE, IT NO, and NL)**	**SURVTOOLS (DK and NL)**
Like	Provision of a conceptual model for integrated surveillance of AMU and AMR surveillance	Comprehensive evaluation of collaboration Participatory evaluation Provision of a clear guidance	Automated analysesProgress monitoringEasy to communicate results	Easy progress monitoring Participatory evaluation Evaluation of the implementation levels	Comprehensive and multi-faceted OH assessment Evaluation of implementation quality	Objectivity Comprehensive framework for different evaluation aspects
Difficulty	No provision of guidance to collect and analyze of data	Evaluation of collaboration only	Why need for such detailed data?	Subjectivity Crude scoring method	Cumbersome	Requirement of training for conducting evaluation Time-consuming for evaluation of complex aspects
Be aware of	Necessary combination with other tools depending on the evaluation question	Characterization and evaluation of integration regarding collaborative objectives and context	Not possible to measure minor progress of epidemiological performance	Complexity in terms of people to include Self-assessment tool Results not comparable across countries	Requirement of training for application Resource demanding	Provision of an evaluation plan only, not AMU and AMR specific
Not covering	Guidance for conducting evaluation	Surveillance performance	Environment and plant sector specifically	One Health assessment Distinction between ongoing and incomplete activities Evaluation of quality of activities	Progress monitoring Surveillance performance	Laboratory aspects One Health assessment

The ECoSur was liked because it allowed evaluation of collaboration in detail; however, the level of abstraction in the language in the existing version of the tool was a struggle. ISSEP was liked because it described the relationship between the integrated surveillance activities for AMU and AMR, OH outputs produced, and the different expected outcomes very well.

NEOH was liked for being comprehensive, multi-faceted, and fit for a transversal analysis of OH initiatives. The main struggle related to NEOH was that it was cumbersome and time-consuming to use. Similarly, SURVTOOLS was liked because information for evaluation of all aspects of a surveillance system including the epidemiological part is provided as scientific references. Furthermore, an epidemiological calculator is provided. However, SURVTOOLS is one of the tools that only provide an evaluation plan.

### Scoring of the Themes Describing the Scope of the Tool

The results from the scoring of each tool for the eight themes describing the scope of the tool in relation to surveillance are shown in Table 6. A summary of the justifications behind the scores are shown in Table 7. A crude summary of the scores for the tools, regarding which themes they covered, showed a limited variation. ATLASS had the highest crude summary scores of 28 followed by ISSEP and ECoSur both with 25.

**Table 6:** Results of scoring of six tools for AMR surveillance evaluation according to eight themes describing the scope of the evaluation tool (the number of times the tool was assessed is given in the bracket).

	**ISSEP (2)**	**ECoSur (2)**	**ATLASS (1)**	**PMP-AMR (4)**	**NEOH (5)**	**SURVTOOLS (2)**
AMU and AMR specific	4	2	4	4	3	2
Collaboration	4	4	4	2	4	2
Resources	2	4	3	3	3	3
Output and use of information	4	3	3	3	3	2
Integration	4	4	3	2	4	2
Governance^*^	3	2	4	4	1	2
Adaptivity	2	4	4	4	3	2
Technical operations	2	2	3	2	2	2
Crude summary score	25	25	28	24	23	17

* *Governance was included in this study by 9 of the 20 of the assessors (however, not a separate theme on the https://guidance.fp7-risksur.eu/welcome/decision-support/). The scoring scale used was 1 = not covered (red), 2 = not well-covered (orange), 3 = more or less covered (yellow), and 4 = well-covered (green)*.

**Table 7:** Synthesis of the underlying reasoning for the scoring according to the eight themes describing the scope of six AMR surveillance evaluation tools.

**Themes**	**ISSEP (CA and UK)**	**ECoSur (DK and VN)**	**ATLASS (DK)**	**PMP (BE, DK, IT, and NO)**	**NEOH (DK, BE, IT, and NO)**	**SURVTOOLS (DK and NL)**
AMU and AMR	Framework developed specifically for AMU and AMR	Not specific for AMU and AMR but can be easily applied for AMU and AMR	Designed for AMU and AMR and residues	Designed for AMU and AMR. Misses components besides farm animals	Not designed for this purpose but can be adapted (e.g., under “objectives of the initiatives”). Most, if not all, of these questions are expected to be included as part of elements 2 and 3	Not developed for AMU and AMR
Collaboration	Allows evaluation of collaboration between the different organizations involved	Collaboration at the heart of the tool, e.g., cross sectors/professions/disciplines/ public/private organizations/geographical/ governance/implementation	Between sectors, all actors, and all levels	Reporting, not data exchange. Participation stakeholders/actors considered for institutions. Gender not considered. Promotes knowledge sharing	Collaboration included in all aspects (in element 1).	No particular guidance; difficult to understand how to evaluate the amount of collaboration
Resources	Questions not included, but data can be collected if economic analysis is part of evaluation	Financial aspects addressed in detail at different levels: planning, allocation, and use	Ask for unlimited or limited budget	Only present in “governance”	Only covered in “planning” and “sharing” aspects of OH-ness evaluation. Focus on allocation: resources to achieve objectives of the initiative (human/physical/financial resources and training). In NEOH handbook, chapter about economic evaluation of OH	Generates a framework for economical evaluation. Epi-calculator available
Output and use of information	Allows to evaluate the outputs of integration and the impacts of integration on decision making and on health and economic outcomes	Allows conclusion about appropriateness of collaborative activities for the expected collaborative outputs (e.g., improving the epidemiological performance). No quantification of impacts on the surveillance value and of costs.	Intermediate-level outputs best addressed	Outputs evaluated (better than impacts), e.g., production of guidelines on prudent use of AM, data reporting to organizations. Not covered in “awareness”	Reveals gaps in OH and where impact of the initiative being evaluated might be improved. Outcomes/impacts depend on type of OH initiative and boundaries of the contextual “system” and resulting ToC. Hence, the evaluator must take into account the appropriate parameters (data and disciplinary paradigms)	If full evaluation, most of the aspects would be covered and impact/output might be possible to measure. Unclear how to measure for intermediate outputs/impacts
Integration	Allows evaluating impacts of integration on decision making/health /economic outcomes	Assessment of the organization and functions of collaboration to achieve the desired level of integration, in coherence with the context	Addressed for many areas, not in-depth	Questions on data reporting, adherence to international testing/data standards/level of knowledge/shared decision making. Not across sectors	Integration measures on many levels, e.g., data integration in organizations, national, regional, or international level, and systems interoperation between different sectors. International testing/data standards not included, unless it is included in “initiative” being evaluated	Not included or advanced to evaluate
Governance	Partly considered when looking at the overall organization/management	Inclusion of many aspects: rationale and objective of collaboration, responsibilities of stakeholders, functionality of governance mechanisms, etc.	Addressed for many areas	Well covered, one main focus of the tool	Partially in the thinking and systemic organization of the OH-ness evaluation. The tool includes consideration of legislation and National Action Plan, if nation is identified as dimensions in the “system”	Not included, but some aspects might be covered if conducting process evaluation
Adaptivity	The tool does not cover this aspect	No monitoring of the progress of collaboration. Monitoring and evaluation of collaboration performance	Measure progress	Designed for measuring improvement	Can be assessed through repeated evaluations. If a dedicated process evaluation is done, the progress can be studied. Evaluator and framework design are “key”	Obtainable if evaluation is done twice (over time) to identify improvement
Technical operations	Includes questions on technical aspects, e.g., sampling/methodology	No evaluation of surveillance performance, even if taken into account evaluation of certain collaboration attributes	Quality of epidemiological designs not covered	Includes questions on the targets of surveillance (e.g., pathogens). Low without ATLASS	Not among evaluation objectives. Include few questions probing for capacities/data handling. Could be part of operations assessment of OH-ness, but extent lies upon evaluator and framework followed	Cover technical efficiency/performance, other laboratory aspects not guided/covered

PMP-AMR, ATLASS, and ISSEP have been developed specifically for AMR-related activities. NEOH and ISSEP were perceived as the best tools for evaluation of all OH aspects, and ECoSur and ISSEP for evaluation of the quality of collaboration. ATLASS is the only tool evaluating laboratory activities specifically. Only ISSEP produced a direct measure of the “integration” and “impact on decision making.” SURVTOOLS has an epi-sample size calculator and is, hence, the only tool providing a quantitative assessment of the technical operations in surveillance.

## Discussion

### Tools Developed Specifically for Evaluating AMU and AMR Surveillance

Only PMP-AMR, ATLASS, and ISSEP have been developed especially for evaluating AMU and AMR surveillance. Generally speaking, ISSEP was the only tool assessed that addressed AMU and integration aspects. The strengths of PMP-AMR and ATLASS are governance and, hence, strategic implementation of NAPs. PMP-AMR addresses neither evaluation of design of surveillance nor integration or collaboration. ATLASS is structured in such a way that detailed information about the sectors involved and the laboratories in the surveillance system can be captured. Thereby, it addresses the gaps in a laboratory's capacity to implement surveillance activities. A quantitative evaluation of the epidemiological designs is impossible in ATLASS. Moreover, ATLASS does not provide an output of the level of integration—but all data collated could provide the evaluator with an impression of the level of integration in the system evaluated.

However, the other evaluation tools were also considered suitable for evaluation of AMU and AMR surveillance programs. In fact, several of the tools showed a high degree of flexibility and were applicable to different surveillance evaluation objectives. Still, the most accurate evaluations originated from the tools that match the specific evaluation questions. Generally speaking, an evaluation of integrated AMU and AMR surveillance systems will benefit from using tools developed specifically for evaluating AMR surveillance and OH aspects since specific characteristics are encountered.

### User Friendliness and Potential Value

The PMP-AMR and ATLASS tools are to a high extent self-instructive and the questions were, therefore, easy to answer. The structure of PMP-AMR was very easy to understand, whereas ATLASS was more complicated to fill in, since it comprises many questions at all levels of organization. The handbook/guidance/surveillance evaluation wiki to SURVTOOLS was perceived by some of the assessors as very clear and easy to read. It also provides advice on how to cover many of the required aspects of evaluation. The online evaluation tool itself looks very aesthetic but covers less information than the handbook and is not fully self-instructive for all evaluation objectives. NEOH requires knowledge of both the relevant context (in the NEOH framework denoted “the underlying system and its system boundaries”) and the integrated surveillance activities (“the initiative under evaluation”) in question, because the assessor must define all components that form part of the underlying system (the context) included in or affected by the surveillance. NEOH allows the assessor to identify and assess expected outcomes based on the ToC of the initiative. ToC is a specific type of methodology for planning, participation, and evaluation that is used in companies, philanthropy, and not-for-profit and government sectors to promote social change. Further, it defines long-term goals and then maps backward in time to identify the necessary preconditions and actions to be taken. The ToC focus will lead to learning and perhaps a better understanding of the surveillance and its potential societal impacts. It is easy to get lost in the extensive handbook published to assist in using NEOH, and a quick guide is currently missing. The many detailed questions about integration such as OH implementation including systemic organization and level of sharing (infrastructure aspects) and learning (operational aspects) allow for nuances in the answers and, thereby, a better quality of the results. However, the evaluator should be aware that applying this tool requires time investment and training, including specific training in “systems thinking.”

ISSEP, ECoSur, and SURVTOOLS also allow for an in-depth analysis requiring collection of more complex data and information. For SURVTOOLS, a specific training in design of epidemiological studies and a wide spectrum of analytical methods are needed before a full exploitation of the tool can be expected. Many of the tools could also be used to guide the design of AMU and AMR surveillance systems in addition to evaluation of existing systems.

Many of the tools, especially ATLASS, produce intermediate outputs of how well the different parts of the program are integrated and how well the partners collaborate. In contrast, the interpretation of evaluation results of ECoSur supports the identification of strengths and weaknesses of collaboration and the formulation of recommendations. Among the six tools investigated, this tool allows for addressing collaboration in most detail and in different dimensions.

It became clear during this study that adequate resources are needed to perform a full evaluation, sometimes requiring involvement of many assessors and/or stakeholders, and it might take weeks to months to finalize. For all tools, training and instructions would be required to understand the tools sufficiently well to work effectively. Furthermore, the assessor should preferably have a moderate level of understanding of surveillance processes. Moreover, it is important to balance the degree of complexity of the evaluation tool with the available resources in terms of number of people, data, and time.

### Output and Use of Information (Impact)

The ISSEP and partly SURVTOOLS approaches provide a conceptual basis for structuring the evaluation of different surveillance outcomes, from the level of integration to the evaluation of the decisions as well as economic efficiency. The outputs of an evaluation may consist of first-level outputs, such as epidemiological performance measures, as well as intermediate outputs, such as how well the system is integrated. For successful AMU and AMR surveillance, the final impact would be that there are antibiotics available to treat future generations of humans and animals against infections. PMP-AMR and ATLASS only produce intermediate outputs through the theme collaboration. It remains unknown whether this and similar themes really reflect what is necessary to implement to reach the final desired impact in the AMU and AMR surveillance. ATLASS and PMP-AMR are contributing to this final impact by providing evaluation of the governance, strategic support, and budgets for surveillance. An evaluation of the impact of surveillance will be further addressed in a phase 2 of the Co-Eval-AMR, just initiated as a follow-up project funded by Joint Programming Initiative for AMR (JPIAMR) (https://www.jpiamr.eu/project/coeval-amr-phase-2/).

### The Limitations of the Study

We have presented the experiences of eight country-based case study groups in using six evaluation tools. Due to resource constraints, some tools were only assessed in a limited number of case studies. Some of the tools were only scored by two assessors, by two assessor groups, or by the creator(s) of the tool. For NEOH, ECoSur, ISSEP, and PMP-AMR, co-developers of the tools were involved in the assessment, but the tools were also assessed by other case study groups. The assessments were done by different persons, and the scores were perceived as crude and subjective. The assessors had varying levels of understanding of the evaluation tools; some were involved in the development of one of the tools, whereas others were trained in using a specific tool. The first group of assessors may have had greater insights into the tool(s) that they assessed and may have been biased in some aspects of the assessment, e.g., user friendliness. During the assessment process, there was some convergence in the scoring done by the assessors due to the development of a common understanding of the words and sentences used in the tools. Therefore, the results of the scoring of the functional criteria had a higher variation than the results of the scoring of the attributes that was done later in the process. The qualitative assessments are probably more informative for the pros and cons of each tool than the actual scores. The remaining tools were assessed by “non-developers.”

Monitoring and stewardship of AMU as part of AMR surveillance were not addressed in the assessment. In the second phase of the Co-Eval-AMR, additional assessments using other tools are planned. Moreover, focus will be on how to assess the impact of integrated surveillance systems for AMU and AMR as well as on how to evaluate governance. The online assessment system made by the Co-Eval-AMR project group can also be used by other scientists for doing similar comparisons and hence more experiences will be collected (https://coevalamr.fp7-risksur.eu/). Most of the participants in the case study groups were veterinarians or professionals working within veterinary public health. Persons in human health only participated indirectly when being interviewed, and there was no focus on the environment. In phase 2 of the project, collaboration among others and with social scientists will broaden the scope and the way of looking at surveillance and evaluations.

### Development of Assessment Methodology and Reporting the Results to Capture the Variation in the Underlying Reasoning

In the Co-Eval-AMR project, the methodology was developed to capture the usability of the tools for evaluation of AMU and AMR surveillance activities in a systematic way, allowing for comparisons between assessors. The assessment methodologies covered aspects known as contributing to controlling AMR, e.g., evaluation of OH aspects, mentioned by for instance Holmes et al. (3). The 11 functional aspects included elements such as user-friendliness and whether the tool meets evaluation needs/produces actionable outputs and the resource needed related to data, manpower, and time. In the second phase of the Co-Eval-AMR project, improvements in assessment criteria will be considered.

As opposed to the other tools, ISSEP and SURVTOOLS generated only a plan for how to conduct the actual evaluation based on the chosen evaluation questions. Hence, scoring these for some of the 11 functional aspects and the eight themes was difficult. The PMP-AMR, ATLASS, ECoSur, and NEOH tools provide semi-quantitative evaluation outputs. PMP-AMR and ATLASS measure the progress over time and can be used repeatedly. Moreover, PMP-AMR and ATLASS seemed suitable for non-scientists too, since they do not require specific knowledge of epidemiology and surveillance for their application. The tools are not interchangeable—they do not have common scopes and objectives; therefore, one cannot choose a tool only based on the appreciation as assessed only by these case studies. Some lack of consistency exists between the work done in the different working groups of the Co-Eval-AMR project, because some of the development of methodologies was undertaken simultaneously in all working groups, e.g., governance was therefore only assessed by “country case study groups” with a few exceptions. The latter reflected in the missing data given as a footnote in Table 3.

### Establishing a Data Capture System for Generation of Assessment Experiences

The developed reporting template enables other assessors to report their experiences using the tools in a comparable way. The template consists of four sections; (1) general information, (2) scoring of 10 functional aspects, (3) SWOT-like approach, and (4) scoring of eight themes describing the scope of the tool. The idea was to develop a kind of user experience scoring overview similar to many internet applications such as TripAdvisor and Google reviews providing the readers with quick, yet detailed, insights of the tools. The template is placed in an online platform on the homepage of Co-Eval-AMR (https://coevalamr.fp7-risksur.eu/). We encourage users of the tools to provide their inputs and expect that over time a growing collection of experiences will help users in choosing more easily among the existing tools.

## Conclusion

Evaluation of integrated surveillance is needed at regular intervals using robust tools. It is important to choose a tool that adequately addresses the specific evaluation objectives. We provided a portfolio of the experiences of 20 users representing eight country-based case studies in which six different tools were applied, to highlight their attributes, pros and cons, and requirements.

Only PMP-AMR, ATLASS, and ISSEP have been developed especially for evaluating AMU and AMR surveillance—with ISSEP being the only tool providing a semi-quantitative score of AMU and AMR integration. All six tools demonstrate a high degree of complementarity. Depending on the evaluation questions selected, assessors may choose among the different tools to conduct the evaluation as such, namely, ECoSur for addressing collaboration, NEOH for the OH-ness and the relationship between ToC and expected outcomes of the surveillance, ATLASS for the laboratory capacities, and SURVTOOL for epidemiological and economic performance.

An online platform for reporting of users' experiences will help users interested in conducting an evaluation of AMU and AMR surveillance in choosing the most adequate tools for their specific evaluation needs: https://guidance.fp7-risksur.eu/welcome/decision-support/. Furthermore, this platform could help further extend general user experience of AMU and AMR surveillance evaluation tools.

## Data Availability Statement

The raw data supporting the conclusions of this article will be made available by the authors, without undue reservation.

## Author Contributions

MS, AH, MB, and LA were substantially involved, and took the lead, in all steps of the study from the conception to the design of the work. AH and UB designed the layout of Table 3 and Table 6. All authors contributed to the assessments and/or to the interpretation of the results of these. Most of the authors initially drafted parts of the paper. All authors approved the final version of the paper. They also agreed to be accountable for all aspects of the work, in ensuring that questions related to the accuracy or integrity of any part of the work were appropriately investigated and resolved.

## Conflict of Interest

MB was involved in the development of ECoSur and LN was involved in the development of NEOH. The remaining authors declare that the research was conducted in the absence of any commercial or financial relationships that could be construed as a potential conflict of interest.
